# Investigating Passive Muscle Mechanics With Biaxial Stretch

**DOI:** 10.3389/fphys.2020.01021

**Published:** 2020-08-20

**Authors:** Benjamin B. Wheatley

**Affiliations:** Department of Mechanical Engineering, Bucknell University, Lewisburg, PA, United States

**Keywords:** muscle stiffness, extracellular matrix, modeling, finite element, non-linear, hyperelastic

## Abstract

**Introduction:**

The passive stiffness of skeletal muscle can drastically affect muscle function *in vivo*, such as the case for fibrotic tissue or patients with cerebral palsy. The two constituents of skeletal muscle that dominate passive stiffness are the intracellular protein titin and the collagenous extracellular matrix (ECM). However, efforts to correlate stiffness and measurements of specific muscle constituents have been mixed, and thus the complete mechanisms for changes to muscle stiffness remain unknown. We hypothesize that biaxial stretch can provide an improved approach to evaluating passive muscle stiffness.

**Methods:**

We performed planar biaxial materials testing of passively stretched skeletal muscle and identified three previously published datasets of uniaxial materials testing. We developed and employed a constitutive model of passive skeletal muscle that includes aligned muscle fibers and dispersed ECM collagen fibers with a bimodal von Mises distribution. Parametric modeling studies and fits to experimental data (both biaxial and previously published) were completed.

**Results:**

Biaxial data exhibited differences in time dependent behavior based on orientation (*p* < 0.0001), suggesting different mechanisms supporting load in the direction of muscle fibers (longitudinal) and in the perpendicular (transverse) directions. Model parametric studies and fits to experimental data exhibited the robustness of the model (<20% error) and how differences in tissue stiffness may not be observed in uniaxial longitudinal stretch, but are apparent in biaxial stretch.

**Conclusion:**

This work presents novel materials testing data of passively stretched skeletal muscle and use of constitutive modeling and finite element analysis to explore the interaction between stiffness, constituent variability, and applied deformation in passive skeletal muscle. The results highlight the importance of biaxial stretch in evaluating muscle stiffness and in further considering the role of ECM collagen in modulating passive muscle stiffness.

## Introduction

The human body is comprised of roughly 40% skeletal muscle – the tissue that drives locomotion, enables fine movements, and provides the capability to breathe in humans and animals alike. This is due to the innate ability of skeletal muscle to generate contractile force and thus drive movement of our musculoskeletal system. While skeletal muscle is a highly adaptable and regenerative tissue (Lieber, 2010; Lieber et al., 2017), neuromuscular conditions such as cerebral palsy, sarcopenia, and damage from acute injury can severely limit the ability of skeletal muscle to function properly (Lieber, 2010). Reductions in contractile capabilities can greatly impair muscle, however more recent work has highlighted the effects of passive muscle stiffness on form and function (Lieber and Fridén, 2019).

Dramatic increases in passive muscle stiffness, for example, can be detrimental for patients with cerebral palsy in comparison to healthy persons (Chapman et al., 2016; Lieber and Fridén, 2019). It follows then that understanding what mechanism(s) and/or constituent(s) in skeletal muscle dictate stiffness is necessary to treat these conditions and prevent extreme impairment. The two constituents that are recognized as the major contributors to the tensile stiffness of passive skeletal muscle are (1) muscle fibers (cells), and (2) the collagenous extracellular matrix (ECM) that provides the hierarchical organization of skeletal muscle (Huijing, 1999; Gillies and Lieber, 2011; Brynnel et al., 2018; Meyer and Lieber, 2018). Passive muscle stiffness has a non-linear and anisotropic nature that has been shown to vary between species and different muscles (Mohammadkhah et al., 2016). It should be noted here that throughout the manuscript we use the term “stiffness” to represent the intricate non-linear, anisotropic, and variable tensile material properties of passive skeletal muscle, and not the structural property *k* often used in Hooke’s Law that characterizes the structural stiffness of a physical object with specific dimensions and material properties.

Uniaxial tensile testing of longitudinal (along-fiber) muscle samples are the most common approach for evaluating tensile stiffness (Calvo et al., 2010; Sato et al., 2014; Lieber and Fridén, 2019). Other efforts to characterize the anisotropy of passive muscle have employed uniaxial stretch in both the longitudinal and transverse (cross-fiber) directions (Morrow et al., 2010; Takaza et al., 2012; Mohammadkhah et al., 2016; Wheatley et al., 2016b). However, during contraction and passive stretch, force is transmitted laterally both within skeletal muscle and between muscles (Huijing, 1999; Ramaswamy et al., 2011; Maas, 2019; Csapo et al., 2020), suggesting that muscle tissue is subject to a multi-axial stress state *in vivo*. This is further supported by the structure of the ECM, which consists of collagen fibrils that are dispersed around the transverse plane (Purslow, 1989; Purslow and Trotter, 1994; Gillies and Lieber, 2011). These observations raise the question as to whether uniaxial stretch is thus the most appropriate *in vitro* experimental technique to evaluate the stiffness of passively stretched muscle, or if multi-axial materials testing may provide certain benefits.

We propose the use of a biaxial tensile deformation as a method to elucidate the passive stiffness of skeletal muscle and have developed and employed both experimental and computational efforts to this end. This method tensions both the longitudinal (along-fiber) and transverse (cross-fiber) orientations simultaneously, which may enact mechanisms that are not observable with uniaxial stretch. Finally, we have previously shown the importance of stress relaxation in modeling passive muscle stiffness (Wheatley et al., 2016a, b), thus time dependence may also provide further insight into muscle stiffness and load sharing between muscle fibers and the ECM.

We also propose the use of computational modeling – in particular finite element analysis (FEA) – to study the passive response of skeletal muscle under both uniaxial and biaxial stretch. We aim to use a continuum-level constitutive model that accounts for stiffness of muscle fibers and the ECM and can capture the variability of stress-stretch behavior that has been observed experimentally (Mohammadkhah et al., 2016). FEA provides a scalable, robust computational tool to simulate skeletal muscle behavior (Jenkyn et al., 2002; Oomens et al., 2003; Blemker et al., 2005; Böl and Reese, 2008). Previous studies include models of muscle at the tissue level (Takaza et al., 2013; Wheatley et al., 2017b), whole muscle level (Blemker et al., 2005; Böl, 2010; Wheatley et al., 2018), with idealized geometries (Jenkyn et al., 2002; Lemos et al., 2008; Chi et al., 2010), and may incorporate the observed anisotropic (Van Loocke et al., 2006; Böl et al., 2014; Pietsch et al., 2014; Mohammadkhah et al., 2016; Wheatley et al., 2016b), hyperelastic (Meyer and Lieber, 2011; Gras et al., 2012; Simms et al., 2012; Wheatley et al., 2016a), and time dependent (Van Loocke et al., 2008; Gras et al., 2013; Wheatley et al., 2016a, c) characteristics of passive skeletal muscle. Thus, FEA is well-suited as a method to explore the tissue-level mechanics of muscle tissue across experimental data sets and loading conditions.

Comprehensively, we aim to explore if experimental and computational efforts to characterize passive muscle stiffness may be enhanced by biaxial stretch by (1) performing planar biaxial materials testing on passive skeletal muscle, (2) developing and employing a robust continuum-level constitutive model of muscle that captures uniaxial and biaxial stress-stretch behavior, and (3) using such a model to explore the similarities and differences between uniaxially and biaxially stretched muscle.

## Materials and Methods

### Experimental Planar Biaxial Testing

**FIGURE 1:**
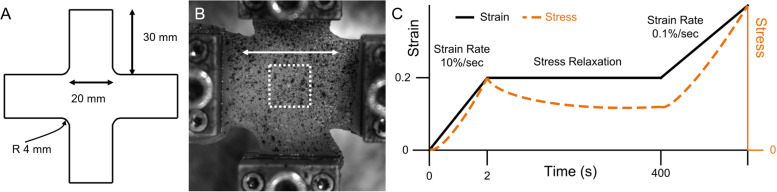
Planar biaxial materials testing overview, with **(A)** cruciform geometry, **(B)** representative planar biaxial sample, where the white arrow denotes the longitudinal direction and the white dashed square denotes approximate DIC region of interest (ROI), and **(C)** experimental stress-relaxation loading protocol schematic (note that axes are not to scale).

Porcine hind limbs were acquired from a local abattoir on the day of sacrifice for testing. Tissue was cooled and stored at 0°C prior to testing. No live animal handling was performed by any participants in this study. A total of four animals, seven muscles, and *n* = 16 total samples were used for testing. The biceps femoris muscle was harvested using standard dissection scalpels. Muscles were sliced along the orientation of fibers with a custom tool that provides 10 mm spacing between the dissection top and a high-profile histology blade (Labus and Puttlitz, 2016). Each ∼10 mm thick sample was then cut into a cruciform shape with a custom cruciform press, aligning the muscle fibers with one cruciform arm (Figure 1). Sample thickness was measured with a caliper mounted on a test stand that was zeroed to the stand platform. Thickness values were recorded in five locations on each sample – in the center of the sample and toward each cruciform arm – and averaged. Mean sample thickness was 8.90 mm with a standard error of 0.29 mm across the five measurements.

All materials testing was performed on a planar biaxial material testing system with 50 lb (∼220 N) load cells. Samples were gripped with 25 mm pyramid grips with an initial spacing of 30 mm between grips. Samples were subject to ten equibiaxial preconditioning cycles of 10% grip-to-grip strain (3 mm) and back to zero at 0.5 Hz prior to testing (Van Ee et al., 2000). A 0.02 N equibiaxial pre-load was then applied immediately prior to testing. The testing protocol included an equibiaxial ramp of 20% nominal (grip-to-grip) strain (6 mm) at 10%/s followed by a hold until 400 s to allow for tissue stress relaxation. Samples were then subject to equibiaxial constant rate stretch at 0.1%/s nominal (grip-to-grip) strain (0.03 mm/s) until failure. Failure was manually identified *post hoc* in stress-time curves where significant (>∼10%) decreases in stress were observed.

Digital image correlation software (Correlated Solutions, Inc.) was used to track strain during the constant rate ramp pull in a ∼10 × 10 mm region of interest (ROI) in the center of the sample. A solid in a reference configuration **X** that undergoes a deformation under an external load is placed into a deformed configuration **x**, which is described by the deformation gradient **F** (Eq. 1). For a 2D problem such as a single camera digital image correlation system, **F** is a 2 × 2 matrix of the deformations relative to orthogonal axes (Eq. 1). From **F**, 2D muscle ROI stretch λ (Eq. 2) can be calculated for the longitudinal and transverse orientations (Szczesny et al., 2012). Nominal (grip-to-grip) stretch was measured directly from grip displacement. Nominal stress *S* was determined by dividing load cell force by the product of sample arm length (30 mm) and mean sample thickness. A linearized modulus $E=\frac{\Delta \,S}{\Delta \,\lambda }$ was calculated from the initial and final points of the constant ramp pull data. For comparative purposes between orientations, we used nominal stress and implemented a finite element model to determine ROI Cauchy (true) stress and material properties. All stress-stretch data were averaged either over time (for stress-relaxation data) or over stretch (for constant ramp pull data) for model fitting.

(1) $$\text{F}=\frac{\partial \text{x}}{\partial \text{X}}=\left[\begin{array}{cc} F_{11} & F_{12}\\ F_{21} & F_{22} \end{array}\right]$$

(2) $$\lambda_{1}=\frac{\left|\partial {\text{x}}_{1}\right|}{\left|\partial {\text{X}}_{1}\right|}$$

### Constitutive Modeling

For a 3D solid subject to an external load, the governing linear momentum balance (Newton’s second law) can be written as Eq. 3 and the governing angular momentum balance can be written as Eq. 4, where σ is the Cauchy (true) stress, ρ is the density of the solid, **b** is the body force vector, and **a** is the acceleration vector. Assuming equilibrium with negligible body force, Eq. 3 reduces to ∇⋅σ = 0. For full derivations and further reading, the reader is directed toward Holzapfel’s *Non-linear Solid Mechanics* (Holzapfel, 2000).

(3) ∇⋅σ+ρ⁢b=ρ⁢a

(4) $$\sigma_{j\,i}=\sigma_{i\,j}$$

**FIGURE 2:**
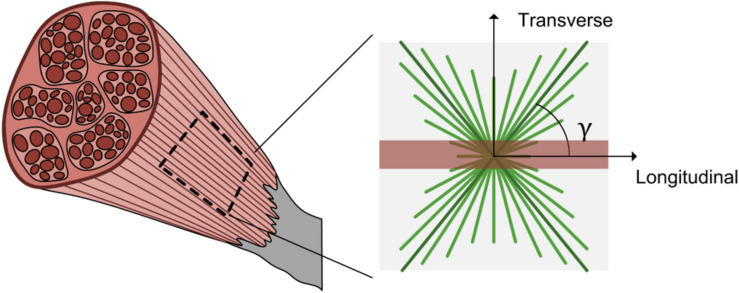
Schematic of passive muscle model for extracellular matrix and muscle fibers. A representative 2D square of muscle tissue shows the longitudinal or muscle fiber direction (red) and two families of collagen fiber dispersion (green) offset from the muscle fiber direction by an angle γ.

The mechanical properties of musculoskeletal soft tissues such as skeletal muscle are often modeled with a strain energy density function, including any specified intricacies such as non-linearity, anisotropy, and nearly incompressibility. To characterize the variable nature of passive muscle anisotropy and non-linearity (Mohammadkhah et al., 2016), we have employed a continuum model Ψ_tot_ that includes contributions of an isotropic ground matrix ${\bar{\text{Ψ}}}_{\text{iso}}$, muscle fibers ${\bar{\text{Ψ}}}_{\text{fibers}}$, the collagenous ECM ${\bar{\text{Ψ}}}_{\text{ECM}}$, and a volumetric response Ψ_vol_ (Eq. 5). As muscle exhibits nearly incompressible behavior (Van Loocke et al., 2006; Takaza et al., 2012), this formulation features a decoupled deviatoric response $\,\bar{\text{Ψ}}={\bar{\text{Ψ}}}_{\text{iso}}+{\bar{\text{Ψ}}}_{\text{fibers}}+{\bar{\text{Ψ}}}_{\text{ECM}}$ and a dilatational response Ψ_vol_. Here deformation is characterized by the volume ratio *J*, the deviatoric right Cauchy–Green deformation tensor $\bar{\text{C}}=J^{-\frac{2}{3}}\,{\text{F}}^{T}\,\text{F}$, the first deviatoric invariant of $\bar{\text{C}}$ denoted by ${\bar{I}}_{1}$, and the deviatoric pseudo-invariant ${\bar{I}}_{4}=\text{m}\cdot \bar{\text{C}}\cdot \text{m}$ that measures the square of muscle fiber stretch whose direction is defined by the unit vector **m** (Holzapfel, 2000). The Cauchy (true) stress σ can then be defined as a function of the constitutive model (Eq. 6, where dev(−) is the deviatoric operator, *p* is hydrostatic pressure and **I** is the identity matrix) (Holzapfel, 2000; Maas et al., 2012). While further detail is provided below regarding specific constitutive relations, the general model employed here is an uncoupled, fiber-reinforced material with two families of fibers – aligned muscle fibers and bimodal, continually distributed ECM collagen fibers (Figure 2; Yousefi et al., 2018; Bleiler et al., 2019). This formulation attributes muscle fibers and the ECM as the main load-bearing constituents in passively stretched muscle (Smith et al., 2019).

(5) $${\text{Ψ}}_{\text{tot}}\,\left(\bar{\text{C}},J\right)={\bar{\text{Ψ}}}_{\text{iso}}\,\left({\bar{I}}_{1}\right)+{\bar{\text{Ψ}}}_{\text{fibers}}\,\left({\bar{I}}_{4}\right)+{\bar{\text{Ψ}}}_{\text{ECM}}\,\left(\bar{\text{C}}\right)+{\text{Ψ}}_{\text{vol}}\,\left(J\right)$$

(6) $$\sigma =\text{dev}\,\left(2\,J^{-1}\,\bar{\text{F}}\,\frac{\partial \bar{\text{Ψ}}}{\partial \bar{C}}\,{\bar{\text{F}}}^{T}\right)+p\,\text{I}$$

The isotropic ground matrix model was modeled with an uncoupled neo-Hookean strain energy density formulation, and the volumetric term with a logarithmic function (Eqs 7 and 8, where *c*_*1*_ is a shear-like modulus and *k* is a bulk-like modulus). Due to the highly anisotropic, non-symmetric, and nearly incompressible nature of skeletal muscle (Van Loocke et al., 2006; Takaza et al., 2012; Mohammadkhah et al., 2016), *c*_*1*_ was selected as a low (but non-zero) constant value for this study and *k* was selected to ensure near-incompressibility, as provided in Table 1.

**TABLE 1:** All model material parameters and units, with fixed values provided and omitted values for parameters that were varied in this study.

Model component	Parameters			
Isotropic matrix ${\bar{\Psi }}_{i\,s\,o}$	*c*_*1*_ = 0.1 (kPa)			
Muscle fibers ${\bar{\Psi }}_{f\,i\,b\,e\,r\,s}$	ξ (kPa)			β (−)
Muscle ECM ${\bar{\Psi }}_{E\,C\,M}$	μ (kPa)	γ (deg)		*d* (−)
Volumetric response *Ψ*_*vol*_	κ = 10,000 (kPa)			
Viscoelasticity	*g*_*i*_ (−)		τ_*i*_ = 0.05, 1, 20, 400 (sec)	

(7) $${\bar{\text{Ψ}}}_{\text{iso}}\,\left({\bar{I}}_{1}\right)=c_{1}\,\left({\bar{I}}_{1}-3\right)$$

(8) $${\text{Ψ}}_{\text{vol}}\,\left(J\right)=\frac{k}{2}\,{\left(\ln J\right)}^{2}$$

The muscle fiber contribution term was defined as a power law to model non-linear stress-stretch behavior of passive muscle when stretched in the direction of muscle fibers (Eq. 9, where ξ is a modulus-like parameter and β is the power parameter) (Takaza et al., 2012; Wheatley et al., 2016b). The ECM strain energy density function defines the behavior of a continually dispersed, 3D bimodal von Mises distribution of tension-only fibers (Ateshian et al., 2009). The formulation presented here is modified from an ellipsoidal bivariate von Mises distribution to describe the anisotropic and inhomogeneous collagen fiber distribution in articular cartilage (Zimmerman and Ateshian, 2019). Due to the continually dispersed nature of the fibers, the strain energy density function is an integration over a unit sphere of volume *V* of the product of the distribution *R*(**n**) (where **n** is the orientation of the ECM collagen fibers) and the fiber constitutive law ${\bar{\text{Ψ}}}_{n}$ (Eq. 10). The distribution *R*(**n**) is further broken into two functions using spherical angle functions *P*(θ) (where θ is the azimuth angle) and *Q*(φ) (where φ is the declination angle) (Eq. 11). If one assumes that the ECM fibers have no directional preference in the transverse plane (perpendicular to muscle fibers), then *P*(θ) decomposes to the circle equation (Eq. 12). The remaining dispersion term, *Q*(φ) describes the ECM collagen fiber dispersion in the along-fiber plane (Figure 2) with a bimodal von Mises function that includes the primary ECM collagen fiber orientation angle γ (the angle of offset from muscle fibers to ECM collagen fibers) and a dispersion term *d* that characterizes the degree of alignment of ECM collagen fibers (Eq. 13). This equation also includes an integration term *q*(*d*,γ) that enforces ∫*R*(**n**)*dV* = 1. By varying the ECM orientation angle γ and the dispersion term *d*, the relative density of ECM collagen fibers can be continuously defined throughout the solid. Finally, a neo-Hookean type fiber constitutive law was used for the ECM collagen fibers (Eq. 14), with a modulus μ that is a function of the square of the collagen fiber stretch ${\bar{I}}_{n}$ (FEBio User Manual 2. 8, 2018). These formulations also assume that fibers (both muscle fibers and ECM collagen fibers) can only sustain tension, not compression (Weiss et al., 1996).

(9) $${\bar{\text{Ψ}}}_{\text{fibers}}\,\left({\bar{I}}_{4}\right)=\frac{\text{ξ}}{\beta }\,{\left({\bar{I}}_{4}-1\right)}^{\beta }$$

(10) $${\bar{\text{Ψ}}}_{\text{ECM}}\,\left(C\right)=\int R\,\left(n\right)\,{\bar{\text{Ψ}}}_{n}\,\left({\bar{I}}_{n}\right)\,d\,V$$

(11) ∫R⁢(n)⁢d⁢V=1=∫P⁢(θ)⁢Q⁢(ϕ)⁢d⁢V

(12) $$P\,\left(\theta \right)={\left[\left(\cos^{2}\theta +\sin^{2}\theta \right)\right]}^{-1/2}$$

(13) $$Q\,\left(\phi \right)=\frac{1}{q\,\left(d,\gamma \right)}\,\left\{\exp\left[2\,d\,\cos^{2}\left(\phi +\gamma \right)\right]+\exp\left[2\,d\,\cos^{2}\left(\phi -\gamma \right)\right]\right\}$$

(14) $${\bar{\text{Ψ}}}_{n}\,\left({\bar{I}}_{n}\right)=\frac{\mu }{4}\,{\left({\bar{I}}_{n}-1\right)}^{2}$$

A quasi-linear Prony series viscoelastic formulation was used to model stress-relaxation of passively stretched skeletal muscle (Wheatley et al., 2016a). Briefly, the deviatoric stress $\bar{\sigma }$ can be defined as a function of a convolution integral (Eq. 15, where *G*(*t*) is the relaxation function, *t* is time, and ζ is an integration variable). A Prony series relaxation function (Eq. 16) enables the use of viscoelastic coefficients *g*_*i*_ and associated time constants τ_*i*_ that characterize the amount and rate of relaxation, respectively. For this study, we fixed τ_*i*_ terms as spaced parameters to ensure a broad range of relaxation rates (Table 1) and varied *g*_*i*_ terms (Vaidya and Wheatley, 2019).

(15) $$\bar{\sigma }\,\left(t\right)=\int_{-\infty }^{t}G\,\left(t-\zeta \right)\,\frac{d\,\bar{\sigma }}{d\,\zeta }\,d\,\zeta$$

(16) $$G\,\left(t\right)=1+\sum_{i=1}^{4}g_{i}\,\text{exp}\,\left(-\frac{t}{\tau_{i}}\right)$$

### Finite Element Modeling

**FIGURE 3:**
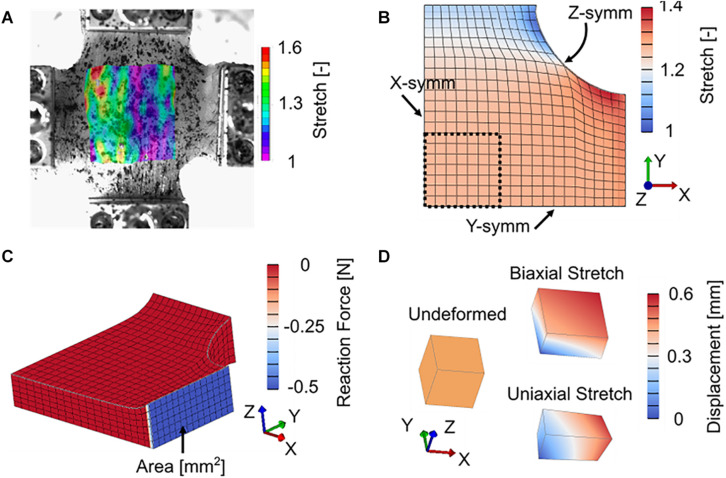
**(A)** Representative color contour plot of longitudinal (horizontal) stretch from digital image correlation. **(B)** Deformed symmetric cruciform finite element model with model ROI (black dashed line) symmetric boundary conditions (note that Z-symm is on the bottom, hidden face), and color contour plot of longitudinal stretch. **(C)** Deformed symmetric cruciform finite element model, showing initial cross-sectional area and reaction force at the model boundary (grip locations) used to determine model nominal stress. **(D)** Undeformed and deformed single element finite element model. Model Cauchy stress was directly output from the single element.

All finite element modeling results presented here were conducted using the open source finite element package FEBio (Maas et al., 2012). A custom plugin was written to apply the bivariate von Mises distribution of the ECM collagen fibers *R*(**n**). To model biaxial stretch, a symmetric, eighth cruciform finite element model consisting of 2,184 linear hexahedral elements was developed (Figure 3B). This model was chosen to represent a cruciform 30 mm × 30 mm in width and height and a thickness of 8.9 mm. In addition to symmetric boundary conditions (Figure 3B), the face of each cruciform arm was fixed to a rigid body and subject to displacements to mimic the experimental protocol (Figure 3C). Reaction force divided by initial arm cross sectional area was calculated as nominal stress and total model length was divided by initial model length to determine nominal stretch. By using the *solid mixture* capabilities in FEBio, separate viscoelastic parameters were assigned to the stress contributions from the muscle fibers and ECM.

Constitutive parameters were optimized to experimental data by fitting model nominal stress to experimental nominal stress. Model ROI stretch was calculated based on the position of model ROI surface nodes similar to experimental stretch (Eqs 1 and 2), then used as a validation to experimental DIC stretch (Figures 3A,B). Parameter optimization was completed in two steps – first the viscoelastic Prony series parameters were fit to normalized stress-relaxation data for both the longitudinal and transverse directions, then hyperelastic parameters were fit to the full set of longitudinal and transverse stress data. This approach has the advantage of reducing the overall number of parameters needed to be optimized at any given step in the process by first determining stress relaxation behavior and then hyperelastic stiffness (Vaidya and Wheatley, 2019). All optimization was performed in MATLAB using constrained non-linear optimization (*lsqnonlin*) by varying model parameters and minimizing the sum of squared residuals between model (σ^*m*^) and experimental (σ^*e*^) stresses as an objective function *obj* across all experimental data points (total number *npts*) (Eq. 17). Nominal stress was used for fitting of our cruciform finite element model (Figure 3C) to planar biaxial experimental data as Cauchy (true) stress cannot be estimated from experimental planar biaxial tests without a correction factor, which is typically determined from FEA.

(17) $$o\,b\,j=\sum_{i=1}^{n\,p\,t\,s}{\left(\sigma_{i}^{e}-\sigma_{i}^{m}\right)}^{2}$$

For comparisons across previously published experimental studies of uniaxial stretch of passive skeletal muscle, a simplified approach of a single linear hexahedral finite element model was implemented (Figure 3D). Three previously published studies of materials testing of skeletal muscle under uniaxial tension were identified (Table 2). These studies provide a range of data across species, muscles, and orientations for model comparison and fitting. The finite element model was fit to the experimental studies by comparing literature Cauchy (true) stress to model Cauchy stress as a function of directional stretch. Data from Wheatley et al. (2016b) were zeroed following an initial stress-relaxation phase for consistency with other data. Following all model fitting, optimized parameters for each data set were used to simulate stress-stretch behavior under uniaxial and equibiaxial stretch. Finally, a simple parameter study was conducted to further highlight the differences in stress-stretch behavior between uniaxial and biaxial stretch. Cauchy stress was used for fitting to previously published uniaxial data and for parametric studies as it requires fewer assumptions to estimate than under biaxial conditions, Cauchy stress is reported in the literature cited here, and the use of Cauchy stress allows for a simplified single element finite element model. No stress conversions were calculated directly from a push forward or pull back operation in this work, as all planar biaxial model fitting used nominal stress only, and all uniaxial and parametric studies used Cauchy stress only.

**TABLE 2:** Summary of data of passively stretched skeletal muscle used in this study.

Study	Species and muscle	Direction tested
Biaxial data – this study	Porcine biceps femoris	Longitudinal and transverse biaxial
Wheatley et al., 2016b	Lapine tibialis anterior	Longitudinal and transverse
Mohammadkhah et al., 2016	Chicken pectoralis	Longitudinal, transverse, and 45°
Takaza et al., 2012	Porcine longissimus dorsi	Longitudinal, transverse, and 45°

To summarize, we fit the cruciform finite element model to planar biaxial data, then used the optimized parameters to simulate uniaxial stretch. Conversely, we fit the simplified finite element model to uniaxial data from three different previously published studies, then used the optimized parameters to simulate biaxial stretch.

### Statistics

Stress relaxation data were normalized to sample peak stress and fit to a power law model (Eq. 18, where σ_*n*_ is normalized stress, *t* is relaxation time, and *a* and *b* are power law coefficients) to characterize the rate of relaxation between orientations. The power law *b* coefficients (rate of relaxation), stress at three time points – peak stress, end of relaxation, and end of ramp pull, and linearized modulus from the pull phase were compared between orientations using a paired *t*-test. A linear regression was performed to investigate the potential effect of post-mortem time on modulus for both directions. For all statistical tests, significance was set at *p* < 0.05.

(18) $$\sigma_{n}=a\,t^{b}$$

Model fits to experimental data were evaluated with an average percent error for each experimental data point, normalized root mean square error (NRMSE, where 1 is a perfect fit and −∞ is the worst possible fit), and root mean square error (RMSE, in kPa) (Vaidya and Wheatley, 2019).

## Results

### Experimental Planar Biaxial Data

**FIGURE 4:**
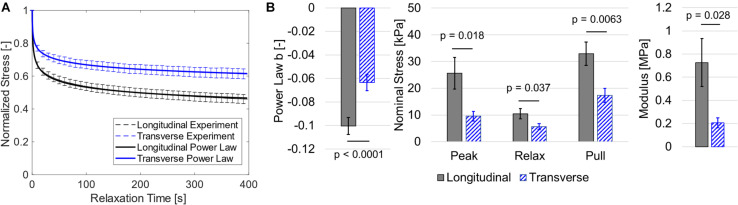
**(A)** Normalized stress relaxation data (shown as dashed mean curves and standard error bars) and a power law fit to the mean data (shown as solid curves). Note that power law fits and experimental data are visually overlapping and thus nearly indistinguishable. **(B)** Bar graphs for mean power law b coefficient, stress data, and linearized modulus with standard error bars.

Biaxial data showed that longitudinal direction nominal stress was greater and decreased at a faster rate during stress relaxation than transverse direction stress. This was supported by both visual analysis of normalized relaxation (Figure 4A) and statistical analysis (Figure 4B). Specifically, the paired *t*-tests suggest that the power law b coefficient was greater in the longitudinal orientation (*p* < 0.0001). Stress was greater at the peak (*p* = 0.021), end of relaxation phase (*p* = 0.037), and end of constant rate pull phase (*p* = 0.0063), and the linearized modulus was greater in the longitudinal direction versus the transverse direction (*p* = 0.028). The power law fits provided excellent agreement to experimental data visually (Figure 4A) and with mean *R*^2^ values of 0.985 and 0.974 for longitudinal and transverse data, respectively. Linear regression results showed that modulus was not correlated with post-mortem time (*p* > 0.6, *R*^2^ < 0.02 for both directions).

### Model Fitting

**FIGURE 5:**
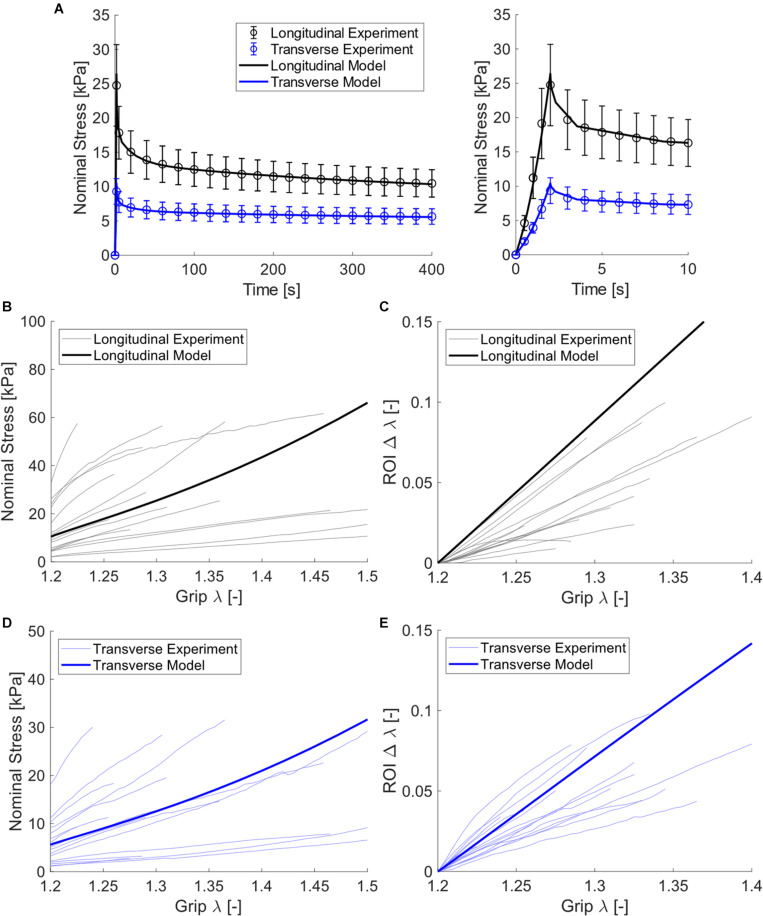
Experimental and model data of passive muscle subject to planar biaxial stretch. **(A)** Nominal stress relaxation step data (open circles with standard error bars) and model fits (solid curves), with the first 10 s of these data for clarity shown at right, **(B)** longitudinal constant rate nominal stress-stretch curves for all experimental samples (thin curves) and model (thick curve), **(C)** longitudinal constant rate ROI stretch-nominal stretch curves, **(D)** transverse constant rate nominal stress-stretch curves, and **(E)** transverse constant rate ROI stretch-nominal stretch curves.

The use of constrained non-linear optimization produced a strong fit of the biaxial finite element model to experimental data, both for the stress relaxation phase as well as the constant ramp pull phase. This is shown both visually (Figures 5A,B,D) and through statistical analysis (NRMSE > 0.9, Table 3). Measured experimental stretch in the sample ROI from digital image correlation and predicted model ROI stretch are provided for model validation (Figures 5C,D). The model showed strong agreement to transverse stretch data, and overpredicted longitudinal stretch somewhat.

**TABLE 3:** Statistical fitting results between model and experimental stress-stretch data.

Data	Mean error (%)	NRMSE (−)	RMSE (kPa)
Biaxial data	0.960	0.959	1.35e-4
Biax stress relax	0.553	0.971	9.22e-5
Biax pull	2.09	0.910	3.91e-4
Wheatley	17.9	0.932	4.67
Takaza	10.7	0.852	4.73
Mohammadkhah	12.0	0.907	1.70

*Data are given in mean percent error between model and experiment, normalized root mean square error (NRMSE), and root mean square error (RMSE) for all data. Biaxial data fits are provided for the overall data set as well as the stress relaxation phase only and the constant rate pull phase only.*

**FIGURE 6:**
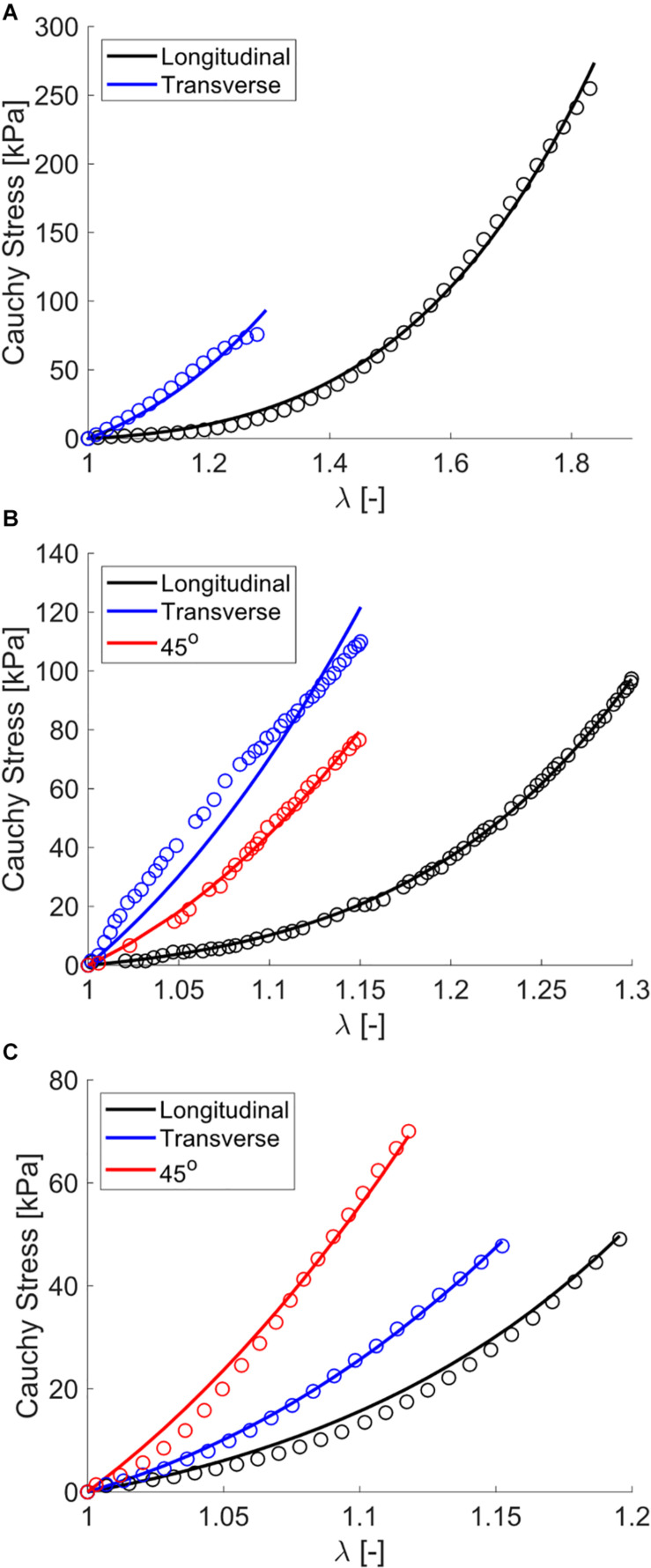
Modeling fits to uniaxial tensile experimental data from the previously published works of **(A)** Wheatley et al. (2016b), **(B)** Takaza et al. (2012), and **(C)** Mohammadkhah et al. (2016). Experimental data are shown as open circles and model data are solid curves.

The single element uniaxial model exhibited strong fitting capabilities across all uniaxial stretch data sets as observed visually (Figure 6) and by evaluating the statistical differences between model outputs and experimental data (NRMSE > 0.85, Table 3). Specifically, the model was able to match a wide range of anisotropy and non-linearity between data sets, including directions of greatest stiffness of the transverse direction (Takaza et al., 2012; Wheatley et al., 2016b) and 45° (Mohammadkhah et al., 2016).

Optimized hyperelastic parameter values (Table 4) demonstrate the variability of passive muscle material properties. For the constitutive model used here, there was a particularly wide range of muscle fiber stiffness ξ (4–110 kPa), ECM modulus μ (28–1,700 kPa), and ECM orientation angles γ (32–90°). It should be noted here that the Mohammadkhah et al. (2016) chicken data best fit produced a negligible muscle fiber modulus, hence the reported value of 0 kPa. Finally, to ensure a unique set of parameters for each optimal fit, the ECM fiber dispersion parameter *d* was fixed for some of the optimizations, as shown in Table 4. Optimized viscoelastic parameter values (Table 5) further highlight the differences in viscoelastic behavior between orientations, as muscle fiber *g*_*i*_ terms were larger than those applied to the ECM term. This shows greater relaxation for the muscle fiber term in comparison to the ECM term.

**TABLE 4:** Optimized parameter values from fits to various experimental data sets.

Data	ξ (kPa)	β (−)	μ (kPa)	γ (deg)	*d* (−)
Biaxial Data	4.20	2.35	28.0	51.1	4*
Wheatley	5.66	2.79	223	90	4*
Takaza	34.7	3.78	1,680	64.3	3.83
Mohammadkhah	0	–	1,400	53.2	8.32

*Asterisk denotes a fixed value of *d=4* due to lack of 45° experimental data.*

**TABLE 5:** Optimized viscoelastic parameters *g*_*i*_ and associated time constants τ_*i*_ from fits to planar biaxial experimental data.

$g_{i}^{\mathrm{fiber}}$ (−)	$g_{i}^{\mathrm{ECM}}$ (−)	τ_*i*_ (sec)
12.9, 2.20, 0.832, 0.979	1.54, 0.634, 0.235, 0.263	0.05, 1, 20, 400

### Modeling Biaxial and Uniaxial Stretch

**FIGURE 7:**
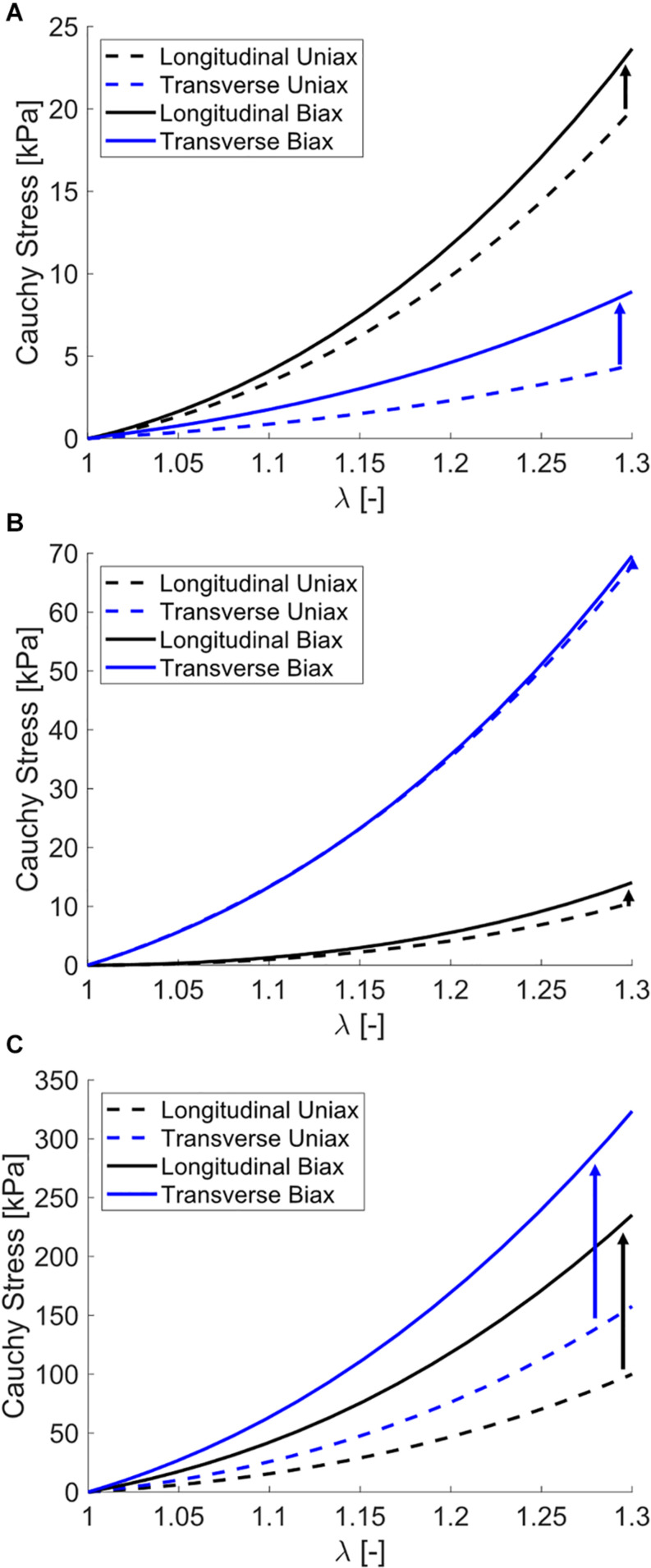
Stress-stretch curves for simulated uniaxial stretch (dashed) and biaxial stretch (solid) for optimized parameters from **(A)** biaxial data presented in this study, **(B)** Wheatley et al. (2016b), and **(C)** Mohammadkhah et al. (2016). The increase in stress-stretch curve stiffness with biaxial stretch versus uniaxial stretch is denoted with arrows. Note that some models predict a negligible increase in stiffness (Wheatley) and others a major increase in stiffness (Mohammadkhah).

**FIGURE 8:**
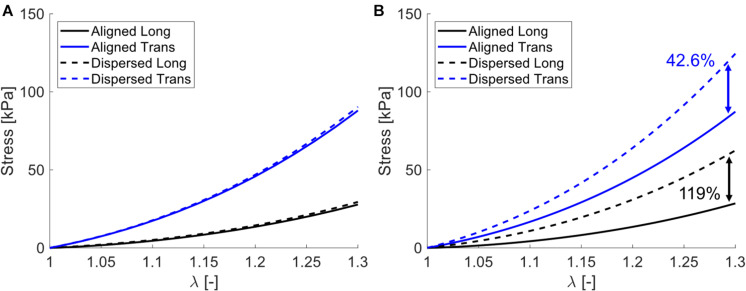
Parametric study stress-stretch curves for **(A)** uniaxial stretch and **(B)** biaxial stretch. Note that for both models (Aligned – solid curves and Dispersed – dashed curves) they exhibit nearly identical uniaxial behavior for both longitudinal (back curves) and transverse (blue curves), but distinctly different behavior when subject to biaxial stretch, with percentage differences between Aligned and Dispersed shown at λ = 1.3.

Simulating both uniaxial stretch and biaxial stretch with each optimized parameter set showed different effects of biaxial stretch on model response (Figure 7 and Table 6). Specifically, stress-stretch curves from Wheatley et al. (2016b). Parameters were largely unaffected by biaxial versus uniaxial stretch, while biaxial stretch greatly increased stiffness for the Mohammadkhah et al. (2016). Parameter set. A parametric study of uniaxial and biaxial stretch for two different parameter sets – one with Aligned fibers and one with Dispersed fibers – shows the models exhibit nearly identical stiffness behavior under uniaxial stretch (Figure 8A) but distinctly different behavior under biaxial stretch (up to 119% difference, Figure 8B). This was observed for both the longitudinal and transverse directions, highlighting the role of the dispersed ECM fibers and assumptions of anisotropy in altering model behavior. Parameter values used (Table 7) fall within those optimized to experimental data (Table 4).

**TABLE 6:** Differences in Cauchy stress at λ = 1.3 between uniaxial and biaxial stretch conditions for all optimized parameter sets.

Output		Biaxial data	Wheatley	Takaza	Mohammadkhah
Longitudinal	–	18.5%	32.2%	92.2%	135%
	kPa	3.69	3.42	89.8	135
Transverse	–	100%	2.47%	24.8%	105%
	kPa	4.45	1.68	86.8	166

*Data are reported at a percent increase and absolute increase in kPa.*

**TABLE 7:** Parameter values for the models shown in Figure 8.

Model	ξ (kPa)	β (−)	μ (kPa)	γ (deg)	*d* (−)
Aligned	130	2.25	275	90	8
Dispersed	0	–	475	60	3

## Discussion

### Planar Biaxial Testing

We have presented here, to the best of our knowledge, the first set of experimental data of passive skeletal muscle subject to planar biaxial stretch. From these data, we determined that the porcine hind limb tissue tested exhibited the following characteristics: (1) faster relaxation for longitudinal samples (Figures 4A,B) and (2) greater stiffness for the longitudinal direction (Figure 4B). For the viscoelastic response, we previously measured greater relaxation rate with greater longitudinal stiffness of muscle tissue (Wheatley et al., 2016b), which agrees with the results seen here. The differences in relaxation rate between orientations suggest two mechanisms that support load in passively stretched skeletal muscle. This observation is supported by ongoing efforts that have shown that passive muscle stiffness in mammals is dictated by both the collagenous ECM (Meyer and Lieber, 2011, 2018) and muscle fibers themselves (Brynnel et al., 2018). Here we suggest that both constituents may contribute to longitudinal stiffness, and that measuring the anisotropic viscoelastic response may further elucidate load sharing between constituents.

It is known that muscle exhibits stress relaxation at both the muscle fiber level (Meyer et al., 2011; Rehorn et al., 2014) and the whole muscle or tissue level (Best et al., 1994; Gras et al., 2013; Wheatley et al., 2016a). Meyer et al. (2011) measured ∼95% stress relaxation when stretching muscle fibers at 2,000%/s and ∼80% stress relaxation at 20%/s, which exceeds stress relaxation observed in highly collagenous tissues such as tendon (Atkinson et al., 1999). Based on these findings and the observation of less relaxation in the transverse direction from our data, the ECM may exhibit less stress-relaxation than muscle fibers. This requires further experimental efforts to confirm or deny, however. To the best of our knowledge, there have been no studies that have compared viscoelastic behavior between single fiber and tissue level samples or have tried to measure the viscoelastic properties of the ECM directly or indirectly. Such a study would help contextualize tissue-level measurements of longitudinal and transverse viscoelastic behavior in regards to the contribution of muscle fibers and the ECM to tissue stiffness.

In comparison to previously published data, most studies of passively stretched muscle have observed a greater stiffness in the transverse direction in comparison to the longitudinal direction, albeit to varying degrees (Takaza et al., 2012; Mohammadkhah et al., 2016; Wheatley et al., 2016b). One previous study observed greater stiffness in the longitudinal direction (Morrow et al., 2010). These comprehensively suggest that anisotropy may be variable in skeletal muscle and may depend on a range of physiological factors. Exploring the link between anisotropy and *in vivo* function was outside the scope of this work but would be appropriate for future studies. We have previously hypothesized that a greater longitudinal stiffness was the result of rigor mortis (Wheatley et al., 2016b), however in this work all testing in this study was completed within seven hours to reduce this risk (Van Ee et al., 2000; Van Loocke et al., 2006) and tissue stiffness was not correlated with post-mortem testing time (*p* > 0.1, *R*^2^ < 0.2). It is thus unlikely that our data are driven by rigor mortis alone. Use of a relaxing agent (Meyer and Lieber, 2018) could be used to further prevent the effects of post-mortem stiffening. Nonetheless, the data presented here should not be viewed as a comprehensive set of muscle material properties, but as a validation of an experimental and computational technique to investigate muscle stiffness.

### Constitutive Modeling of Experimental Data

Fitting results show the capability of our constitutive model to accurately simulate the range of experimentally observed anisotropic and non-linear stress-stretch behavior. This is shown both visually (Figures 5, 6) and through statistical evaluation (Table 3). We also used the experimental biaxial ROI stretch data for model validation (Figure 5). These results comprehensively suggest that our model is well-suited for studying the tissue-level mechanics of passively stretched skeletal muscle. To encourage a unique solution for each data set, the ECM fiber dispersion parameter *d* was fixed based on a qualitative comparison to muscle ECM fiber dispersion (Purslow and Trotter, 1994). Additionally, dataset characteristics such as viscoelasticity (biaxial data), data at 45° (Mohammadkhah and Takaza), and non-linear longitudinal data (Wheatley) enforced a unique solution for each optimization.

The constitutive model used in this study includes a non-linear muscle fiber term ${\bar{\text{Ψ}}}_{\text{fibers}}\,\left({\bar{I}}_{4}\right)$ which is a function of ${\bar{I}}_{4}$, the square of muscle fiber stretch. We chose to employ a power law function for this term as it models the non-linear stress-stretch response of muscle in the longitudinal orientation (Mohammadkhah et al., 2016; Wheatley et al., 2016a) with only two parameters. The optimized values for the modulus-like parameter ξ (0–108 kPa) and for the power coefficient β (2.35–3.78) are reasonable, although ξ = 0 for the Mohammadkhah data is questionable. However, Mohammadkhah et al., 2016 data was obtained from chicken pectoralis muscle tissue, which as they note has a higher collagen content (Nishimura, 2010). This may partially explain why our model optimization approach identified a negligible muscle fiber modulus for this data set if collagen is dominating the stress-stretch response. Our remaining muscle fiber modulus-like values of 4.2–108 kPa compare reasonably to experimental observations of ∼40 kPa in mice (Meyer and Lieber, 2018).

Our model of muscle ECM ${\bar{\text{Ψ}}}_{\text{ECM}}\,\left(\bar{\text{C}}\right)$ describes the collagen fibers with a neo-Hookean hyperelastic model (with a shear modulus μ) and a bimodal von Mises distribution (with angle γ and dispersion *d*). Our use of a single modulus term is a simplification of a highly complex combination of ECM collagen amount, type, crosslinking, and crimp. While these each have been studied in regards to tissue stiffness through either experimentation (Smith and Barton, 2014; Chapman et al., 2015; Mohammadkhah et al., 2018; Lieber and Fridén, 2019; Smith et al., 2019) or modeling (Gindre et al., 2013; Bleiler et al., 2019; Spyrou et al., 2019; Valentin and Simms, 2020), developing a unique set of parameters from healthy tissue-level data that incorporates each of these was outside of the scope of this work. This also does not address the different layers of ECM structure such as perimysium and endomysium. We instead chose to use the approach of minimizing the number of model parameters while ensuring a strong fit to experimental data.

Purslow and Trotter (1994) measured muscle ECM collagen fiber orientations under a range of physiological conditions and found that the primary fiber alignment angle was dependent on stretch, but ranged from ∼20–80°. Qualitatively, recent mammalian ECM scanning electron microscopy by Sleboda et al. (2020) found that multilayered, collagen-rich ECM was common between a range of species but that microstructure was less consistent. These studies suggest that ECM fiber angle and dispersion may vary with a range of mechanical, anatomical, and physiological factors such as animal size and muscle fiber type distributions. The optimized ECM fiber angles we determined (32–90°) thus seem reasonable.

In considering specific modeling studies relevant to this work, Yucesoy et al. (2002) modeled the muscle fibers and ECM as distinct but linked constituents. Gindre et al. (2013) developed a microstructural model of a muscle fiber wrapped with a single family of dispersed ECM fibers to explore titin and ECM contributions. Yousefi et al. (2018) showed how a similar model of the ECM as the only load bearing mechanism with two perfectly reinforcing fiber directions can describe the observed anisotropy in passively stretched bovine, porcine, and chicken muscle. Bleiler et al. (2019) designed and formulated a passive constitutive model with dispersed collagen fibers surrounding muscle fibers that could be integrated into a finite element simulation. Teklemariam et al. (2019) used a similar micromechanical approach with distinct muscle fiber and ECM domains. Spyrou et al. (2019) developed a multiscale model that employed homogenization from a microstructurally derived model to a continuum-level response.

While each of these studies present advantages for modeling the passive response of skeletal muscle, we have chosen to use a similar approach to Yousefi et al. (2018) with the extension of the model to include ECM collagen dispersion and muscle fiber stiffness. After applying assumptions for the low-stiffness isotropic ground matrix (Wheatley et al., 2017a) and near-incompressibility (Takaza et al., 2012), this model required five parameters to describe the hyperelastic response – two for the muscle fibers (stiffness and non-linearity) and three for the ECM (stiffness, direction, and dispersion). The advantage of this approach is a relatively low number of parameters while still enabling model robustness. The use of a Prony series viscoelastic model may increase the overall number of parameters of the model, but as we have shown in this and previous works (Vaidya and Wheatley, 2019), those parameters can be optimized with a two-step fitting procedure. Based on stress-stretch data alone, it would be unclear how load is shared between the ECM and muscle fibers. However, the stress-relaxation data shows distinct time dependent differences between longitudinal and transverse stress relaxation rate (Figure 4). This suggests load may be supported by both muscle fibers and ECM, and perhaps more so the muscle fibers in the longitudinal direction.

It should be noted that the model chosen here enables a wide range of stress-stretch behavior and is generally informed by muscle physiology, but is not derived from microstructure and does not account for effects of interaction between the ECM and muscle fibers. The parameters (such as ECM fiber angle and dispersion) may be generally related to tissue microstructure, but are not direct analogs. One must be careful not to conclude concrete microstructural findings based on the fitting results presented here.

### Modeling Uniaxial Versus Biaxial Stretch

Expanding our modeling from fitting to simulations of uniaxial versus biaxial stretch showed variability between data sets (Figure 7 and Table 7). Generally speaking, materials exhibit greater stiffness when stretched biaxially versus uniaxially. However, for highly anisotropic materials with multiple families of fibers, the effect may not be as dramatic as expected, as shown in the uniaxial versus biaxial comparisons of the Wheatley et al. (2016b) parameter set (Figure 7B and Table 6). In this case, the model ECM fibers are aligned perpendicular to muscle fibers and have low dispersion and during biaxial stretch each set of fibers are recruited independently. Conversely, the Mohammadkhah et al. (2016) parameter set increased in excess of 100% in both the longitudinal and transverse directions (Figure 7C and Table 6). Here the ECM fibers are oriented between directions and highly dispersed, which recruits these fibers during both longitudinal and transverse stretch. Thus, the biaxial deformation will stretch the ECM fibers to a greater amount.

The potential physiological relevance of a case where biaxial stretch and uniaxial stretch exhibit similar stress-stretch behavior can be seen in Figure 8 and Table 7. Here we have identified two sets of parameters that fall within the previously optimized values that have nearly indistinguishable uniaxial stress-stretch behavior in both the longitudinal and transverse orientations (Figure 8A). When subject to biaxial stretch however, Dispersed shows drastic changes in stiffness while Aligned is largely unaffected (113% difference in the longitudinal direction between models). This presents a simplified case where two muscles that may seem to have the same mechanical properties when stretched uniaxially would in fact have quite different mechanics when subject to a more complex deformation. In effect, these differences are “hidden” by uniaxial stretch. This could partially explain that differences in longitudinal stiffness between cerebral palsy and healthy muscle cannot be explained by collagen content, quantity, and cross-linking alone (Chapman et al., 2015; Lieber and Fridén, 2019; Smith et al., 2019).

Smith et al. (2019) discuss these collagen content-passive stiffness correlations and a relatively minor contribution of collagen crosslinking that this observation “… suggests the intriguingly possibility that higher-order structures may determine tissue stiffness to a greater extent than molecular components.” We suggest that ECM collagen fiber orientation and dispersion may be these “higher-order structures” and show with our model how differences in tissue stiffness could be hidden by uniaxial stretch (Figure 8A). As noted above, the technique employed here is a continuum-level hyperelastic constitutive model. We do not imply that this model is a direct prediction of tissue microstructure, only that our model has shown robust and accurate stress-stretch behavior and that similar mechanisms may be present.

Another consideration for uniaxial versus biaxial stretch is the observation of transverse load transmission in contracting muscle as well as laterally between individual muscles (Huijing, 1999; Yucesoy et al., 2008). If load generated longitudinally by muscle fibers is transmitted transversally through the ECM, then muscle tissue must be subject to a multi-axial stress state *in vivo*. Our parametric study suggests that biaxial stretch could enact a stiffening effect to longitudinal stress-stretch response in comparison to uniaxial only (Figures 8A,B). For tissues that exhibit higher load sharing of the ECM, this effect could be exaggerated, and biaxial stretch would in effect increase the perceived tissue stiffness, and thus perhaps increase the efficiency of load transfer *in vivo* during contraction. However, further experimental research is needed to confirm if this is the case for biaxially stretched skeletal muscle. Nonetheless, we have highlighted the importance of a biaxial deformation in passively stretched skeletal muscle, and hope that this consideration can drive future work to better understand load transmission *in vitro* and *in vivo*.

### Limitations and Future Directions

This work is not without limitations. Firstly, the geometry selection of a simplified, idealized cruciform or single element cuboid is clearly not a representation of the geometric/structural complexities of whole, *in vivo* skeletal muscle. However, the experimental data used in this study are generated from tissue samples isolated from whole muscle, and thus do not represent a full *in vivo* environment either. This isolation is necessary to accurately determine tensile material properties. While a more detailed set of geometric finite element models could be developed to match average specimen geometry from each uniaxial experiment, this may not necessarily yield improvements in fit or different study conclusions. The advantage of our geometric approach is in computational efficiency and simplicity – as the optimization protocol that fit the model to experimental stress-stretch curves does not require significant computation time and is highly stable. Nonetheless, as experimental and finite element models of *in vivo* muscle deformation have shown complex strains (Blemker and Delp, 2005; Böl et al., 2015), use and validation of this model in such cases would be a significant benefit to the field.

While our constitutive model exhibits robustness in simulating tensile stress-stretch behavior (Figure 6), it does not model microstructural and physiological characteristics such as collagen crosslinking, multiple collagen types, or muscle fiber-ECM interactions. Including such components would likely yield increased robustness and physiological accuracy of such a model. However, our model has exhibited efficacy in simulating a wide range of passive muscle stretch. We have shown that this model can inform future studies of ECM structure – such as collagen fiber orientation and dispersion – while fitting tissue-level data and maintaining experimental observations such as near-incompressibility.

It should be noted that model validation across uniaxial and biaxial stretch would strengthen future applications of this model. Additionally, experiments such as biaxial materials testing coupled with decellularization or muscle fiber isolation would provide necessary insight into the extent to which this model or future improved models can accurately characterize load sharing between muscle fibers and the ECM. This would greatly strengthen this work, and provide strong efficacy for application of this model to *in vivo* conditions of muscle impairment such as cerebral palsy. We have also not explored the model response under compression or during active contraction as those are outside the scope of this work. Thus, this model should be viewed not as a comprehensive model of passive skeletal muscle, but as an effective tool in better understanding passive muscle stiffness.

## Conclusion

Based on section “Results and Discussion” of this work, we have made the following observations, recommendations, and conclusions:

(1) We performed biaxial stress-relaxation testing on passive skeletal muscle and suggest that this approach can be used to effectively characterize passive muscle mechanics.
(2) Our model of a dispersed ECM contribution and aligned muscle fibers was able to exhibit broad variability in simulating and fitting tensile stiffness, non-linearity, and anisotropy of passive skeletal muscle.
(3) This model, in conjunction with experimental data, exhibited the role of biaxial stretch in measuring passive muscle stiffness and suggesting future work to explore inconsistent correlations between muscle ECM collagen measurements and passive stiffness.

Future validation, development, and employment of modeling and biaxial experimentation would elucidate the role of the ECM in *in vivo* muscle function, and help explain how detrimental changes to muscle stiffness – such as those observed in cerebral palsy – may be explained by ECM structure.

## Data Availability Statement

The datasets generated for this study are available on request to the corresponding author.

## Author Contributions

BW contributed to the study design, experimentation and modeling, data processing, and manuscript development.

## Conflict of Interest

The author declares that the research was conducted in the absence of any commercial or financial relationships that could be construed as a potential conflict of interest.
